# Comparison of treatment with insulin detemir and NPH in women with gestational diabetes mellitus: glycemic control and pregnancy outcomes. A retrospective study

**DOI:** 10.1007/s42000-023-00490-2

**Published:** 2023-09-29

**Authors:** Paraskevi Kazakou, Stavroula A. Paschou, Marina Mitropoulou, Vasiliki Vasileiou, Vasiliki Sarantopoulou, Eleni Anastasiou

**Affiliations:** 1 Endocrine Unit and Diabetes Center, Department of Clinical Therapeutics, Alexandra Hospital, School of Medicine, National and Kapodistrian University of Athens, Athens, Greece; 2 Department of Endocrinology, Alexandra Hospital, Athens, Greece

**Keywords:** Gestational diabetes, Detemir, NPH

## Abstract

**Purpose:**

The objective of this retrospective study was to compare glycemic control, pregnancy outcomes, and neonatal outcomes in women with gestational diabetes mellitus (GDM) treated with (a) insulin detemir and (b) insulin neutral protamine Hagedorn (NPH).

**Methods:**

A total of 192 women with GDM were included in the analysis. Ninety-eight women received detemir, while 94 women received NPH. Data regarding medical history, glycemic control, and time and mode of delivery, as well as neonatal outcomes, were recorded.

**Results:**

Baseline characteristics were comparable between the two groups. There were no differences with respect to the week of insulin initiation, total insulin dose, duration of insulin therapy, daily insulin dose/weight in early and late pregnancy, or the number of insulin injections per day**.** Maternal overall weight gain during pregnancy and weight gain per week did not differ either. The detemir group had slightly lower HbA1c levels at the end of gestation [median: det 5.2% (33 mmol/mol) vs NPH 5.4% (36 mmol/mol), *p*=0.035). There were no cases of hypoglycemia or allergic reactions in the two groups. There were also no differences regarding neonatal outcomes according to the available data, given that data in some cases were missing.

**Conclusion:**

The use of insulin detemir was found to be equally effective and safe compared to NPH in women with GDM.

## Introduction

Hyperglycemia during pregnancy is associated with increased risk of adverse fetal, neonatal, and maternal outcomes, which can be avoided by tight glycemic control [1, 2]. The Hyperglycemia and Adverse Pregnancy Outcomes (HAPO) Study has shown that there is a continuous association between the rise of maternal glucose levels and the risk of adverse pregnancy outcomes, including neonatal hypoglycemia and increased rate of macrosomia and cesarean section [3]. Several studies have demonstrated that in GDM, these complications can be reduced by achieving better glycemic control [4, 5]. However, this still remains a challenging and sometimes unattainable goal. During pregnancy, only human insulin was widely used until recently, but its use is now limited due to the risk of hypoglycemia. Better pharmacokinetic and pharmacodynamic profiles of insulin analogs may help overcome this obstacle.

In recent years, insulin detemir, a long-acting insulin analog, has been increasingly studied for use in pregnancy. In 2012, insulin detemir received US Food and Drug Administration approval for reclassification to pregnancy category B from pregnancy category C. This was based on the results of small observational studies and, mostly, of a randomized control trial (RCT) comparing insulin detemir and NPH in 310 pregnant women with type 1 diabetes mellitus (T1DM) [6–9]. This large prospective study found that insulin detemir was not inferior to NPH concerning glycemic control as well as maternal and neonatal outcomes [8, 9].

Pharmacodynamics defines the main difference between the two types of insulin. NPH peaks between 4 and 12 h after injection with a duration of action of around 14 h, while insulin detemir is characterized by a slower onset and a longer duration of action (18-20 h) with no pronounced peak [10, 11]. NPH has been studied and used for several years in non-pregnant and pregnant patients with proven safety and efficacy. In contrast, it cannot mimic the physiological profile of insulin release and has been associated with higher rates of maternal hypoglycemia [12]. On the other hand, detemir as an insulin analog is newer on the market and less studied. Furthermore, it has a “peakless” action profile, with lower variability of action, and can lead to fewer hypoglycemic events [13, 14].

However, the number of studies investigating GDM women on insulin detemir is limited. In a randomized controlled trial, 87 pregnant women with GDM or type 2 diabetes mellitus (T2DM) received insulin detemir (*n*=42) or NPH (*n*=45), with short-acting insulin aspart as needed. The primary outcome was overall mean blood glucose (BG) during the insulin treatment. The trial found no difference in the primary outcome, namely, the time it took to achieve good glycemic control, maternal weight gain, and perinatal/neonatal outcomes in the two treatment arms. Hypoglycemic events were lower in the detemir group [15]. Another randomized trial by Ji et al. compared the efficacy and safety of insulin detemir versus NPH in 132 women with pregestational diabetes and 108 with GDM and found that detemir was able to control blood glucose and reached the targets faster and more effectively, with lower incidence of maternal hypoglycemia and comparable adverse birth outcomes [16]. In a recent multinational prospective cohort study, “The Real World EVOLVE Study,” in pregnant women with pre-existing diabetes, insulin detemir was associated with a similar risk to other basal insulins of major congenital malformations perinatal or neonatal death, hypoglycemia, preeclampsia, and stillbirth [17]. Furthermore, a meta-analysis of RCTs comparing insulin detemir versus NPH in 1450 pregnant women with gestational or pregestational diabetes showed significant results in favor of insulin detemir concerning maternal hypoglycemic events and gestational age at delivery [18].

Notably, real-life data concerning the use of insulin detemir alone in GDM is limited. The aim of this retrospective study was to compare glycemic control and pregnancy outcome as well as fetal and neonatal outcomes between women with GDM treated with insulin detemir and insulin NPH.

## Methods

### Patients

We retrospectively searched the database of the Diabetes Center in Alexandra Hospital, School of Medicine, National and Kapodistrian University of Athens, Athens, Greece, specifically for the following women: (1) who presented GDM (total number 952); (2) who were treated with insulin detemir or NPH; (3) without a need for rapid-acting insulin; and (4) for a period of 30 months (from January 2013 to July 2015). GDM was diagnosed based on a positive 75-g 2-h glucose tolerance test using the IADPSG (International Association of Diabetes and Pregnancy Study Groups) criteria [19]. Treatment with insulin was initiated based on the following: fasting blood glucose>90 mg/dl (5.0 mmol/l), 1-h postprandial glucose>130 mg/dl (7.4mmol/l) (more than 30% of measurements in 1 week), and/or evidence of macrosomia (FAC >75^th^ percentile) or polydramnios on fetal ultrasound [20]. A total of 192 women with GDM were finally included. Of these, 98 women received detemir and 94 women received NPH. The study was carried out in accordance with the International Code of Medical Ethics of the World Medical Association (Declaration of Helsinki). Approval and informed consent were obtained from the Alexandra Hospital ethics committee.

### Protocol

We retrospectively analyzed data from pregnant women who had previously visited our clinic for routine prenatal care. We reviewed their recorded measurements, including weight, HbA1c, blood pressure, fasting plasma glucose, glucose, and 1-h glucose after a standard breakfast offered in the clinic, as well as insulin dose. The pregnant women had visited our clinic at approximately 2-week intervals. All women had been advised to follow a specific diet and to perform self-monitoring of plasma glucose values 4-6 times daily, specifically, morning fasting, 1 h after meals, before bedtime, and before main meals. The patients had recorded their blood glucose values in diabetes diaries, which were evaluated at each visit. Insulin doses had been adjusted to maintain good glycemic control according to treatment targets (fasting blood glucose < 90mg/dl (5 mmol/l), that is, 1-h postprandial blood glucose ≤130mg/dl (7.8 mmol/l) [20]. Fetal ultrasound had been performed on a routine basis at inclusion, at 13, 21, and 32 weeks, and whenever necessary.

Adverse events were also recorded (allergies and hypoglycemias). Major hypoglycemia was defined as an event in which a person was unable to treat herself on her own. Minor hypoglycemia was defined as an episode in which the subject was able to treat herself and had blood glucose of <56mg/dl (3.1 mmol/l) [8]. Hypoglycemic episodes were recorded by the patients in their diaries. Preeclampsia was also recorded. Data regarding medical history, parameters of glycemic control, insulin type, insulin dose, number of injections, adverse events, time and mode of delivery, and neonatal outcomes were identified from the medical records retrospectively. Neonatal outcomes included any congenital malformation, preterm delivery (delivery <37 completed weeks), early fetal death (<22 completed weeks), perinatal death (death occurring between 22 completed weeks and 1 completed week after delivery), neonatal mortality (postpartum death after 7 completed days and before 28 completed days after delivery), 5-min Apgar score <7, neonatal hypoglycemia after delivery (plasma glucose<45mg/dl (2.5 mmol/l)), neonatal jaundice, time of delivery, cesarean delivery, birth weight, birth weight adjusted for gestational age, and live-born infants with a birth weight <10^th^ or >90^th^ percentile for gestational age and sex, according to local practice.

### Statistical analysis

Descriptive results of continuous variables are expressed as mean ± standard deviation or median [interquartile range (IQR)]. Distribution and homogeneity of the variables were tested with the Shapiro-Wilk test. Differences in continuous variables between groups were assessed using Mann-Whitney tests or Student’s t-tests. For the comparison of proportions, the chi-square and Fisher’s exact tests were used. ANCOVA was used in order to examine the difference in final HbA1c between the two groups after adjusting for age, BMI, weight gain, and initial HbA1c. Α value of *p*<0.05 was considered statistically significant. Statistical analyses were performed using SPSS statistics 23.0 (SPSS, Chicago, IL, USA).

## Results

A total of 192 pregnant women were retrospectively included in the study; 98 were treated with insulin detemir and 94 with NPH during pregnancy. The selection of the type of insulin therapy was made in the context of everyday clinical practice and was based mainly on the consideration of cost and the need not to be restricted exclusively to one manufacturer. Patient demographics and baseline characteristics are shown in Table 1. The baseline characteristics, such as age, BMI before pregnancy, rates of smoking, hypertension, education level, ethnicity and family history of T2DM were comparable between the two groups.

**Table 1:** Demographic and baseline characteristics

	Detemir group (*n*=98)	NPH group (*n*=94)	*p* value
Age (years) (mean±SD)	36 ± 4.1	36.8 ± 5	0.350
Prepregnancy BMI (kg/m^2^) (median, IQR)	27.4 (24.9-33.1)	26.4 (22.9-30.9)	0.111
Ethnicity (Greek), n* (%)	87/98 (88.8)	76/93 (81.7)	0.168
Education (higher), n* (%)	38 (39.2)	19 (22.1)	0.076
Smoking, n* (%)	16/95 (16.8)	14/87 (16.1)	0.892
Family history of T2DM, n* (%)	42/98 (42.9)	39/90 (43.3)	0.947
Hypertension, n* (%)	0/98 (0)	0/94 (0)	-

^*^Data are missing in a few cases.

The statistical tests used are Pearson’s chi-squared test and Fisher’s exact test

Maternal weight gain per week from the start of insulin until delivery and, overall, in pregnancy did not differ between the groups (Τable 2). There were no episodes of symptomatic hypoglycemia or allergic reactions in both groups. Furthermore, no hypertensive disorders or preeclampsia were noted.

There were also no differences regarding the week of insulin initiation or the duration of insulin therapy (Table 2). The median daily insulin dose (IU/kg) was comparable between the detemir and NPH groups both at the beginning of insulin treatment and at delivery (Table 2). In addition, there were no differences with respect to the number of insulin injections per day (given as a single bedtime injection in all cases).

**Table 2:** Maternal outcomes

	Detemir group (*n*=98)	NPH group (*n*=94)	*p* value
Overall weight gain (kg)	10.3 (6.5-14.3)	12.4 (6.4-16.8)	0.193
Weight gain per week (g) (Insulin start to delivery)	40 (-18 - 250)	110 (-80 - 250)	0.389
Initial HbA1c (%) (mmol/mol)	5.3 (5.1- 5.6) 34 (32- 38)	5.4 (5.1-5.6) 36 (32 - 538)	0.626
Final HbA1c (%) (mmol/mol)	5.2 (5-5.5) 33 (31 - 37)	5.4 (5.2-5.6) 36 (33 - 38)	0.035
Week of insulin initiation	28 ± 7	27 ± 7.5	0.428
Treatment duration (days)	53 (35 - 73)	56.5 (42.5- 78.5)	0.446
Initial daily insulin dose/weight (ΙU/kg)	0.1 (0.1 - 0.2)	0.1 (0.1 - 0.2)	0.299
Final daily insulin dose/weight (ΙU/kg)	0.14 (0.09 - 0.18)	0.13 (0.1 - 0.16)	0.829
Fasting BG (mg/dl) (mmol/L) (initial)	104 ± 11.1 (5.7± 0.6)	104.7± 9.4 (5.8± 0.5)	0.726
Postprandial BG (mg/dl) (mmol/L) (initial)	144 ± 38.0 (7.9 ± 2.1)	133.8 ± 31.1 (7.4 ± 1.7)	0.130
Fasting BG (mg/dl) (mmol/L) (Final)	94.9 ± 10.9 (5.3± 0.6)	93.2 ± 8.1 (5.2± 0.45)	0.406
Postprandial BG (mg/dl) (mmol/L) (Final)	114.6 ± 14.9 (6.4 ± 0.8)	113.5 ± 13.5 (6.3 ± 0.7)	0.687

The statistical tests used are the Mann-Whitney test and Student’s t-test. Results are reported as mean±SD or median, IQR".

The mean fasting and mean postprandial BG levels (Table 2) as well as the median HbA1c at the beginning of insulin treatment were comparable between the detemir and NPH groups (Table 2). Moreover, there was no difference between the detemir and NPH groups with respect to the mean fasting and mean postprandial glucose values (Table 2). Regarding HbA1c at the end of gestation, the detemir group presented a slightly lower although significant level compared to the NPH group (Table 2). The difference remained significant after adjusting for age, BMI, weight gain, and initial HbA1c (ANCOVA, *p*=0.007) (Table 3).

**Table 3:** ANCOVA results for final HbA1c as dependent variable

	Type III sum of squares	df	Mean square	F	P
Corrected Model	0.105	5	0.021	59.50	<0.001
Intercept	0.102	1	0.102	288.15	<0.001
Initial HbA1c	0.099	1	0.099	278.72	<0.001
Age	0.000	1	0.000	0.02	0.891
Prepregnancy BMI	0.000	1	0.000	0.27	0.605
Weight gain per week (insulin start to delivery)	0.001	1	0.001	4.20	0.042
Group	0.003	1	0.003	7.68	0.007
Error	0.062	176	0.000		
Total	96.341	182			

Logarithmic transformations were used for the dependent variable.

Data regarding neonatal outcomes were not available. Based on the available data, we found no statistically significant differences between the two groups. Specifically, the median time of delivery, the rate of cesarean section, the rate of preterm delivery, the mean birth weight, the mean birth weight adjusted for gestational age, the median Apgar score, the rate of macrosomia (weight >90^th^ percentile), and the rate of SGA cases (<10^th^ percentile) (0% in both groups) were comparable between the detemir and NPH groups (Table 4). All babies were born alive with no major or minor malformations or episodes of severe hypoglycemia.

**Table 4:** Neonatal outcomes

	Detemir group (*n*=98)	NPH group (*n*=94)	*p* value
Time of delivery (weeks)	38 (37 - 39)	38 (37 – 39)	0.540
Cesarean section, n* (%)	23/37 (62.2)	26/54 (48.1)	0.188
Preterm delivery, n* (%)	5/37 (13.5)	13/62 (21)	0.352
Birth weight (g) (median, IQR)	3182 (2940-3680)	3105 (2820-3445)	0.081
Birth weight adjusted for gestational age (median, IQR)	50 (40-72.5)	50 (40-60)	0.133
Macrosomia, n* (%)	2/36 (5.6)	0/62 (0)	-
Small for gestational age (SGA), n (%)	0/36 (0)	0/62 (0)	-
Apgar score	9 (8-9)	9 (8-9)	0.561

^*^Data are missing in some cases.

The statistical tests used are Fisher’s exact test and Mann-Whitney test.

## Discussion

Information about insulin detemir in GDM is scarce and originates mainly from studies on pregestational diabetes, T1DM, or T2DM or both pregestational and gestational diabetes [6–9, 15–18, 20–26]. To our knowledge, our study is the first to investigate exclusively insulin NPH versus detemir in a peer cohort of women with real GDM diagnosed from the 24^th^ to 28^th^ week of gestation. Insulin detemir was found to be equally effective compared to NPH with no differences observed with respect to maternal adverse outcomes or neonatal complications. In our retrospective cohort, all women received a single bedtime injection of NPH or detemir per day in order to correct fasting BG, without any additional prandial insulin. Postprandial BG was controlled with an appropriate gestational diabetes diet.

Traditionally, improvements in glycemic control during insulin therapy are associated with weight gain [27]. In our study, there were no statistical differences concerning maternal weight gain (both per week and throughout pregnancy) between the two groups. These results are in agreement with most other observational cohorts or RCTs comparing the use of detemir versus NPH in pregnancies with T1DM, T2DM, or GDM [8, 15, 16]. Only one recent RCT in pregnant women with T2DM by Bartal et al. found that insulin detemir was associated with less weight gain compared to NPH [22].

In our cohort, no cases of hypoglycemia were recorded in either group, this probably due to the fact that there was no need for intensification of insulin treatment. Insulin detemir or NPH was injected once daily, while there was no need for rapid-acting insulin. In two observational studies investigating insulin NPH versus detemir in pregnancies with T2DM or GDM, the rate of hypoglycemia between the two groups was comparable [21, 24]. However, in the RCT by Herrera et al. in 87 pregnant women with GDM and T2DM, NPH was associated with a higher risk of hypoglycemia compared to insulin detemir. In line with this, there are other studies investigating detemir versus NPH in pregnancies with pregestational and gestational diabetes, possibly due to the intensive insulin regimen including short-acting insulins [15, 16, 22].

Furthermore, no hypertensive disorders were recorded in either group. Previous studies comparing insulin detemir versus NPH in pregnancies with T1DM, T2DM, or GDM have shown comparable rates of hypertension [9, 16, 21, 24]. Only the aforementioned RCT in pregnant women with T2DM observed lower adverse maternal outcomes, including hypertensive disorders, in the detemir group compared to the NPH group [22].

No allergic reactions were recorded in our study in the two treatment arms. According to most previous studies, adverse drug reactions were rare and similar between the NPH and detemir groups [16, 21, 22, 24]. Notably, in the study by Mathiesen et al., only eight women of the 152 of the detemir group reported adverse events relating to injection sites [8]. In addition, in the study by Herrera et al., a higher rate of allergic reactions in women treated with insulin detemir was noted, forcing them to switch to an alternate medication [15].

We found no differences between the two groups with respect to mean fasting and postprandial BG levels at the beginning of insulin treatment and at the end of gestation. These findings are similar to those of previous observational and RCTs comparing detemir and NPH in pregnancy [15, 26].

In our study, the detemir group presented a slightly lower HbA1c level at the end of gestation, confirmed by the ANCOVA test after adjusting for age, BMI, weight gain, and initial HbA1c. However, Hba1c is not considered as the gold standard marker for assessing glycemic control during pregnancy due to altered red blood cell kinetics, increased erythropoiesis, and hemodilution. Mean BG levels during pregnancy are more sensitive to changes in glucose control over the short term that these patients are treated for. In addition, the RCT of Ji et al. comparing NPH versus detemir in 240 pregnant women with pregestational and gestational diabetes showed that in the early period of treatment, detemir performed better than NPH in terms of controlling blood glucose, as fasting and postprandial BG levels after 1 week of treatment were lower in the detemir group and the time to reach target was shorter. Furthermore, insulin detemir was at least as effective as NPH in a long-termtreatment as after 3 months, HbA1c between the two groups being shown to be similar [16].

In agreement with the study by Mathiesen et al., we found no differences between the two groups in relation to the duration of insulin treatment or the daily insulin dose [8]. Only in the study by Ji et al. was the total insulin dose in the detemir group higher than in the NPH group [16]. By contrast, in our study insulin doses between the two groups were comparable after adjustment for weight.

Overall, there were no statistically significant differences in neonatal outcomes including birth weight, birth weight adjusted for gestational age, percentage of macrosomia, or SGA and rates of preterm delivery or cesarean section, although the relevant data were unavailable. There were no congenital malformations or cases of severe neonatal hypoglycemia, which suggested that insulin detemir was as good as NPH in terms of safety. We acknowledge the lack of data of our study regarding perinatal parameters, such as induction rates and complications in labor. Although, indeed, one limitation might be that some data are missing, our results are consistent with the findings reported by most previous studies [9, 15–17, 21, 24]. Only the recent RCT by Bartal et al. reported lower rates of adverse neonatal outcomes, but with a higher rate of LGA in the detemir group [21].

To the best of our knowledge, this is the largest retrospective study investigating exclusively insulin detemir versus NPH, either of these administered in a single dose at night, in pregnancies with GDM only. In addition, one notable strength of the study is that it was performed in real-life routine conditions among unselected patients in a single center by the same medical team, where the glycemic goals for treatment were constant throughout the study period. The majority of the patients were of Caucasian origin and insulin therapy was initiated taking into consideration the increase of the fetal abdomen circumference on ultrasound.

Limitations of the study are the retrospective nature of the design and the selection of patients, the missing data regarding neonatal outcomes, and the selection of the insulin type. However, the current study provides real-world data, which are very important and are complementary to information derived from RCTs. Moreover, most of the women were of the same ethnicity with a mean prepregnancy BMI under 30 kg/m^2^. Outcomes from this study should be applied carefully in cases of more serious GDM where an intensive insulin regimen is needed, and especially in pregnancies with T2DM.

In conclusion, this study provides evidence that insulin detemir and NPH are equally effective and safe with respect to glycemic control and total insulin dose needed, maternal outcomes and complications, and neonatal outcomes. The only significant difference, though minor, was observed in relation to final HbA1c. The lower HbA1c in the detemir group seems not to have clinical significance, as evidenced by the absence of differences with regard to maternal adverse outcomes or neonatal complications, between the two GDM groups. Finally, the only differentiating factor appears to be the cost of the two options, where NPH is the more economical one.

## Data Availability

Data are available on request from authors.
